# The level of RECQL1 expression is a prognostic factor for epithelial ovarian cancer

**DOI:** 10.1186/s13048-014-0107-1

**Published:** 2014-11-26

**Authors:** Yoko Matsushita, Yoshihito Yokoyama, Hidemi Yoshida, Yuki Osawa, Makito Mizunuma, Tatsuhiko Shigeto, Masayuki Futagami, Tadaastu Imaizumi, Hideki Mizunuma

**Affiliations:** Department of Obstetrics and Gynecology, Hirosaki University Graduate School of Medicine, 5-Zaifu-cho, Hirosaki, Aomori 036-8562 Japan; Department of Vascular Biology, Institute of Brain Science, Hirosaki University Graduate School of Medicine, 5-Zaifu-cho, Hirosaki, 036-8562 Japan

**Keywords:** Ovarian cancer, RECQL1, siRNA, Apoptosis

## Abstract

**Background:**

The human RECQ DNA helicase family is involved in genomic stability. Gene mutations of RECQL2, RECQL3, and RECQL4 are associated with genetic disorders and induce early aging and carcinogenesis. Although previous studies have reported that the level of RECQL1 expression is correlated with the prognosis of some of malignancies, the function of RECQL1 is not yet clarified. The present study aimed to examine the relationship between prognosis and the level of RECQL1 expression in epithelial ovarian cancer (EOC), and to identify the role of RECQL1 in EOC cells.

**Methods:**

The level of RECQL1 expression was determined immunohistochemically in 111 patients with EOC who received initial treatment at Hirosaki University hospital between 2006 and 2011. Effects of RECQL1 on cell growth or apoptosis were examined in vitro using wild-type and OVCAR-3 cells (RECQL1(+) cells) and similar cells transfected with *RECQL1* siRNA transfected (RECQL1(−) cells).

**Results:**

The level of RECQL1 expression was not related to histological type, clinical stage, or retroperitoneal lymph node metastasis, but the expression level was significantly higher (P = 0.002) in patients with recurrence than those without recurrence, and progression-free survival and complete response rate to chemotherapy were also improved in patients with RECQL1-low expression (n = 39) stage III/IV EOC (P = 0.02 and P <0.05 vs RECQL1-high expression patients (n = ), respectively). A cell proliferation and colony formation assays revealed significantly less growth of RECQL1(−) cells compared to RECQL1(+) cells. A flow cytometry using annexin V -FITC and propidium iodide (PI) staining revealed a significant increase in apoptotic RECQL1(−) cells. Cell cycle analysis showed a significantly greater distribution in subG1 phase indicating apoptotic cells in RECQL1(−) cells than in RECQL1(+) cells.

**Conclusions:**

These results suggest that RECQL1 is a prognostic factor for EOC and that RECQL1 contributes to potential malignancy by inhibiting apoptosis.

## Background

Epithelial ovarian cancer (EOC) is the world’s most lethal gynecological cancer and the World Health Organization Global database listed EOC as the seventh leading form of cancer in women in 2008 [1].

Although the mean 5-year survival rate for EOC has improved significantly over the past 30 years, the prognosis remains poor, with a 46% 5-year survival rate [2]. The prognosis for EOC is closely related to the clinical stage of the cancer at diagnosed. The mean 5-year survival rate in advanced stages (FIGO stage III or IV) is as low as 11% to 41% [2]. More than 70% of EOC is detected in the advanced stages mainly because of a lack of early warning signs and of reliable diagnostic tests. Cytoreductive surgery followed by adjuvant chemotherapy is recommended as the primary treatment for advanced EOC. Postoperatively, the combination of a taxane and carboplatin is used as first-line chemotherapy. EOC is highly responsive to initial anticancer treatment, but approximately half of the advanced cases recur within two years and result in poor prognosis due to a decreased response to chemotherapy [3]. Therefore, new clinically useful biomarkers and new targets for treatment of EOC need to be identified so as to initiate intensive treatment.

Initially found in Escherichia coli, RECQ helicase affects the recombination of DNA, and deletion of RECQ helicase causes genomic instability [4]. In addition, deletion of a RECQ helicase SGS1 induces genomic instability in yeast and shortens its life by increasing the sensitivity to drugs that react with DNA [5]. There are five types of RECQ helicase in humans: RECQL1, WRN, BLM, RTS, and RECQ5 [6]. WRN, BLM, and RTS are involved in genetic disorders associated with genomic instability and a high incidence of cancer [7-9]. However, the function of RECQL1 in regulation of cancer growth is not fully clarified. A recent study has indicated that RECQL1, a typical member of the RECQL family [10] unwinds DNA and plays a role in chromosomal stability [11]. Earlier studies have shown a relationship between the level of expression or mutation of RECQL1 and the prognosis for pancreatic cancer, liver cancer, and head and neck cancer [12-14]. More recent study has suggested a significant role of RECQL1 as a proliferative marker in EOC [15].

The present study examined the relationship between prognosis and the level of RECQL1 expression in EOC. This study also identified the role of RECQL1 in cancer cells.

## Methods

### Subjects and tissue samples

An immunohistochemical examination was performed retrospectively on 111 EOCs and 10 normal ovaries samples (5 of proliferative phase and 5 of secretary phase) from patients treated at the Hirosaki University Hospital between 2006 and 2011. Written informed consent had been obtained from all subjects. One slide from each case was reviewed by a gynecologic pathologist (M.F.) to confirm the diagnosis of EOC. The tissue specimens included 45 serous adenocarcinomas, 17 endometrioid adenocarcinomas, 30 clear cell adenocarcinomas, 15 mucinous adenocarcinomas, and 4 other types of carcinomas. All patients were primarily treated with cytoreductive surgery and adjuvant paclitaxel and carboplatin (TC) chemotherapy (paclitaxel 175 mg/m^2^ and carboplatin AUC 6). They received 6 to 9 cycles of this combined regimen. Seventy-one of the 111 patients underwent a staging laparotomy including a retroperitoneal lymphadenectomy. Optimal debulking surgery was achieved in 96 (86.5%) out of the 111 patients if defined as macroscopic residual tumor under 1.0 cm at the end of primary surgery, while the remaining 15 patients had a residual tumor of greater than 1.0 cm in diameter. The breakdown for stages of EOC consisted of 57 patients with stage I EOC, 8 with stage II, 42 with stage III, and 4 with stage IV. The duration of follow-up ranged from 21 to 92 months (median, 64 months). The mean age of patients with EOC at surgery was 55 years old (range, 22 to 84 years old). Clinical and pathological features are shown in Table 1. The acquisition of tissue samples was approved by the institutional review board of Hirosaki University’s Graduate School of Medicine.

**Table 1:** **Patients characteristics**

**Clinico-pathological factors**	**Number of patients**	**%**
Age (Median, range) yr	55 (22–84)	
≤ 40	12	10.8
> 40	99	89.2
Histological type		
Serous adenocarcinoma	45	40.5
Endometrioid adenocarcinoma	17	15.3
Clear cell adenocarcinoma	30	27.0
Mucinous adenocarcinoma	15	13.5
Others	4	3.7
FIGO stage		
I/II	65	58.6
III/IV	46	41.4
Retroperitoneal lymph node involvement		
Negative	57	51.4
Positive	14	12,6
Missing	40	36.0
Residual tumor		
< 1.0 cm	96	86.5
≥ 1.0 cm	15	14.5

### Immunohistochemistry

**Figure 1:**
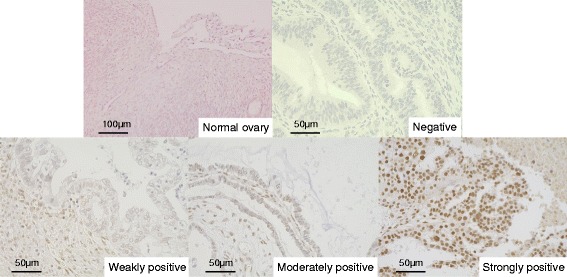
**Staining intensity of RECQL1 in nuclei.** No expression of RECQL1 was found in the normal ovarian tissue. The staining intensity in EOC was graded as negative, weakly positive, moderately positive, or strongly positive. Photomicrographs are shown at 100x or 200x.

The detection of RECQL1 was performed using polyclonal antibody produced in rabbits (Santa Cruz Biotechnology, Santa Cruz, CA). All surgical samples obtained for immunohistochemistry were fixed in formaldehyde and embedded in paraffin. Sections 6 μm thick were routinely passed through xylene and a graded ethanol series. Sections were incubated with antibody overnight 4°C after antigen retrieval in a sodium citrate buffer. Slides were incubated with biotinylated species-specific secondary antibodies for 20 min and then exposed to avidin - biotinylated enzyme complex (VECTASTAIN® Elite ABC KIT, Vector Laboratories, Burlingame, CA). All antibodies were detected using 0.02% chromogen diaminobenzidine (DAB) and they were counterstained with hematoxylin. The level of RECQL1 expression was graded using staining scores, and these scores were calculated by multiplying the intensity of nuclear staining by the stained areas within the tumors. As shown in Figure 1, the staining intensity of nuclei was classified as negative (score 0), weekly positive (score 1), moderately positive (score 2), or strongly positive (score 3). Stained areas were graded as follows: a score of 0 was given to a specimen with an area of 0% staining, a score of 1 was given to a specimen with an area of ≥1% to <25% staining, a score of 2 was given to a specimen with an area of ≥25% to <50% staining, and a score of 3 was given to a specimen with an area of ≥50% staining. For each patient, a slide specimen was observed in a 0.75-mm^2^ field of vision using a 20× objective lens. The mean score from 3 different sites was used as the staining score for that patient. The level of RECQL1 expression was evaluated by 2 researchers (Y.Y. and T.S.) who were not apprised of any physical and clinical information. A staining score of 9 was defined as RECQL1-high expression. Other staining scores were defined as RECQL1-low expression.

### Cell line and cell cultures

OVCAR-3 was obtained from the American Type Culture Collection (Rockville, MD). This cell line is derived from human ovarian papillary adenocarcinoma and was grown in RPMI 1640 supplemented with 10% FCS at 37°C in a water-saturated atmosphere with 5% CO_2_/95% air. OVCAR-3 was verified in writing as being ovarian in origin. OVCAR-3 overexpresses RECQL1 [15].

### siRNA and RNA interference

siRNA (21 bp) targeting RECQL1 mRNA (RECQL1-siRNA) and negative (non-silencing) control siRNA were chemically synthesized using FlexiTube GeneSolution GS5965 for RECQL (QIAGEN, Tokyo, Japan). Cells were transfected with RECQL1-siRNA for 48 hours at a final concentration of 10 nM siRNA using the Hyperfect transfection system (QIAGEN) in accordance with the manufacturer’s instructions. All siRNA sequences had overhanging-3′-TT. siRNA sequences were as follows:

RECQL1 siRNA sense sequence (5′-CAUUGAUCUCUCUUAUGGAtt -3′),

RECQL1 siRNA antisense sequence (3′-ggGUAACUAGAGAGAAUACCU -5′),

control siRNA sense sequence (5′-AGGUCGAGAUGACAUGAAAtt -3′), and

control siRNA antisense sequence (3′- ggUCCAGCUCUACUGUACUUU -5′).

### Real-time quantitative PCR

Total RNA was extracted from cells using an Illustra RNAspin Mini RNA Isolation Kit (GE Healthcare, Piscataway, NJ). Total RNA (5 μg) served as a template for single-strand cDNA synthesis in a reaction using an iScript Advanced cDNA Kit (Bio-Rad, Hercules, CA) under previously described conditions [16] with slight modifications. A CFX96 real-time PCR detection system (Bio-Rad) was used for quantitative analyses of RECQL1 and glyceraldehyde-3-phosphate dehydrogenase (GAPDH). Primer sequences were as follows:

RECQL1-F (5′-GCTGTGGATGAAGTTCACTGCTG-3′),

RECQL1-R (5′- GGAACTGCCGCTTTAAGATACCAA-3′)

GAPDH-F (5′-CCTCCCGCTTCGCTCTCT -3′), and

GAPDH-R (5′- GCTGGCGACGCA AAAGA -3′)

The primers were used at a concentration of 300 nM. Amplification conditions were as follows: 30 seconds at 95°C, followed by 95°C for 5 seconds, and 60°C for 30 seconds for 40 consecutive cycles. After amplification, a melting curve from 65°C to 95°C at 0.5°C increments and 5 seconds per step was generated with continuous monitoring of fluorescence. CFX manager Version 2.1 software (Bio-Rad) was used to create the melting curves and quantitatively analyze data. The level of RECQL1 mRNA in a sample was normalized to that of GAPDH mRNA.

### Western blot analysis

Cell lysates (50 μg protein) were prepared from OVACAR-3 cells transfected with RECQL1-siRNA or control siRNA (as control), electrophoresed through a 12.5% sodium dodecyl sulfate polyacrylamide gel, and blotted as described previously [16]. The protein concentration was determined using Bradford’s method. The blots were probed with the following diluted antibodies for 2 hours: RECQL1 (Santa Cruz Biotechnology) at 1:1000 and β-actin (Sigma-Aldrich, St Louis, MO)) at 1:2000. The membranes were then incubated for 1 hour with the appropriate biotinylated secondary antibodies, transferred to avidin-biotin-peroxidase complex reagent, and incubated in this solution for 30 minutes. DAB was used as a substrate.

### Cell count

OVCAR-3 cells (1 × 10^4^ cells) were cultured in 6-well plates as described in Cell line and cell culture. Cell counts were performed 24 hours, 48 hours and 72 hours after OVACAR-3 cells were transfected with RECQL1-siRNA or control siRNA (as control). To distinguish live and dead cells, cells were stained with 0.3% trypan blue solution (Wako Pure Chemical Industries, Osaka, Japan). Cells were counted in a hemocytometer. The cell count was performed in triplicate, and the total cell count and the number of dead cells were represented as averages.

### Colony formation assay

Single-cell suspensions (50 cells) of OVCAR-3 in RPMI1640 (10% FBS) were seeded in 6-well plates. OVCAR-3 cultures were fixed in 6% glutaraldehyde and stained with 0.5% crystal violet at day 7 [17]. The stained cultures were analyzed by microscopy, and aggregates containing 50 or more cells were counted as colonies. The number of colonies per well was counted. This assay was performed in triplicate.

### Determination of apoptotic cells

Apoptotic or necrotic cells were determined using flow cytometry with an Annexin V-FITC kit (Beckman Coulter, Fullerton, CA). Cells were harvested and washed with cold PBS. Cells were re-suspended with 100 μL annexin-V binding buffer and then incubated with 10 μL annexin-V for 15 min at room temperature in darkness. Four hundred μL binding buffer containing 5 μL propidium iodide (PI) was added to the cells and cells were incubated on ice for 15 min. The cells were applied to a FACS Calibur-500 flow cytometer within 1 hr of preparation. A total of 1 × 10^4^ cells were analyzed in each sample. Evaluation was done 24 hours after transfection of RECQL1-siRNA or control siRNA. The results were interpreted as follows: Cells that were annexin V(−)/PI(−) (lower left quadrant) were considered as living cells; annexin V(+)/PI(−) (lower right quadrant) as apoptotic cells; annexin V(+)/PI(+) (upper right quadrant) as necrotic cells; and annexin V(−)/PI(+) (upper left quadrant) may be bare nuclei, cells in late necrosis, or cellular debris. Experiments were repeated in triplicate.

### Cell cycle analysis

Cell cycle was determined using flow cytometry with a Cell cycle phase determination kit (Cayman Chemical, Ann Arbor, MI). For the cell cycle assay, 1 × 10^5^ cells were seeded in 6-well plate. Evaluation was done 24 hours after transfection of RECQL1-siRNA or control siRNA. Cells were harvested and fixed gently by adding 75% ethanol and placing at −20°C for 4–16 h. The cells were washed twice with PBS, resuspended in 300 μl PBS containing 100 μg/ml PI and 0.1 mg/ml RNase, incubated for 30 min at room temperature in dark, and analyzed using a flow cytometer. FlowJo 7.1.0 software (Tree Star, Ashland, OR) was used for data analysis and at least 10,000 cells were counted for each measurement. Experiments were repeated in triplicate.

### Statistical analysis

All statistical analyses were performed using SPSS (version 21, SPSS Inc., Chicago, IL, USA). Differences in staining scores due to clinico-pathological factors were analyzed using a chi-square test. Progression-free survival (PFS) was defined as the period from the end of the first-line regimen to initiation of the second-line regimen after signs of recurrence were verified in imaging. Overall survival (OS) was calculated from the date of the start of treatment to the date of death or last follow-up. Cumulative survival curves were estimated using the Kaplan-Meier method. Survival curves were compared using the log-rank test. Variables with effect on PFS in univariate analysis were included in the Cox proportional hazard regression model. Multivariate logistic regression was used to calculate hazard ratios (HRs) and 95% confidence intervals (95% CIs) after controlling simultaneously for potential confounders. Variables considered in the models were age, histological types, residual tumor, lymph node status and RECQL1 expression status. Other differences were analyzed by Student’s *t*-test. Statistical significance was set at P <0.05.

## Results

### Relationship between clinicopathological factors and the level of RECQL1 expression

**Figure 2:**
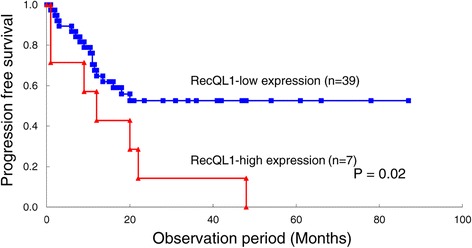
**Comparison of the prognosis between RECQL1-high and RECQL1-low patients with stage III/IV EOC.** There were 7 RECQL1-high patients and 39 RECQL1-low patients. There was significant difference in PFS between RECQL1-high (Median PFS; 12.0 months) and RECQL1-low patients (Median PFS; 18.0 months) (HR; 0.26, 95% CI; 0.38-0.70, P = 0.02).

Immunohistochemistry showed that RECQL1 was not expressed in the celomic epithelium, epithelium lining inclusion cyst or the stroma of the normal ovaries (Figure 1). Of the 111 EOCs, 12 were RECQL1-high expression. No significant correlation was noted between the level of RECQL1 expression and clinical stage, histological type, and retroperitoneal lymph node metastasis. However, the level of RECQL1 expression was significantly higher (P = 0.002) in patients with recurrence than in those without recurrence (Table 2). PFS and OS did not differ significantly for RECQL1-low and RECQL1-high patients when all stages of disease were analyzed (data not shown). PFS of stage III/IV diseases was significantly longer in RECQL1-low patients (n = 39) than RECQL1-high ones (n = 7) (Figure 2, HR; 0.26, 95% CI; 0.38-0.70, P = 0.02), but OS did not differ significantly for the 2 groups (data not shown).

**Table 2:** **Relationship between clinico-pathological factors and RECQL1 expression**

**Factors**	**Number of patients**	**RECQL1- high expression**	**P value**
Stage			
I/II	65	5 (7.7%)	
III/IV	46	7 (15.2%)	Not significant
Histological type			
Serous adenocarcinoma	45	7 (15.6%)	
Endometrioid adenocarcinoma	17	1 (5.9%)	
Clear cell adenocarcinoma	30	1 (3.3%)	
Mucinous adenocarcinoma	15	2 (13.3%)	
Others	4	1 (25%)	Not significant
Retroperitoneal lymph node metastases (n = 53)			
(−)	46	1 (2.2%)	
(+)	7	1 (14.3%)	Not significant
Relapse			
(−)	73	5 (6.8%)	
(+)	38	7 (18.4%)	P = 0.002

Furthermore, univariate and multivariate analyses were performed in relation to PFS and clinico-pathological factors including RECQL1 expression status in stage III/IV EOC. Univariate analysis showed a significantly close association of high RECQL1 expression with PFS, and then, multivariate analysis suggested that high RECQL1 expression as well as a greater residual tumor could be a prognostic factor for PFS (Table 3).

**Table 3:** **Univariate and multivariate analyses for determining prognostic factors of progression free survival in stage III/IV epithelial ovarian cancer**

**Variables**	**No.**	**Univariate**			**Multivariate**		
		**HR**	**95% CI**	**P value**	**HR**	**95% CI**	**P value**
Age							
> 40 (n = 4) vs. ≤40 (n = 42)	46	3.44	0.59-1.76	0.59	2.56	0.33-19.56	0.364
Histological type							
Serous (n = 29) vs. non-serous (n = 17)	46	1.96	0.84-1.09	0.47	1.96	0.20-1.28	0.152
Residual tumor							
< 1.0 cm (n = 32) vs. ≥1.0 cm (n = 14)	46	1.12	0.50-1.05	0.34	0.36	0.14-0.90	0.031
Lymph nodes status							
Negative (n = 16) vs. Positive (n = 14)	30	2.64	0.46-1.42	0.10			
RECQL1 expression status							
High (n = 7) vs. Low (n = 39)	46	0.26	0.38-0.70	0.02	0.35	0.14-0.86	0.022

Abbreviation: No., number of the patients; HR, hazard ratio; CI; confidence interval.

Multivariate analysis was adjusted for age, histological types, residual tumor and RECQL1 expression status except for lymph nodes status because number of the patients who underwent lymph node dissection was insufficient.

### Relationship between the level of RECQL1 expression and the sensitivity to anticancer drugs

The sensitivity to anticancer drugs was evaluated in 46 patients with stage III/IV disease. Complete remission (CR) was defined as disappearance of lesions on diagnostic imaging along with a negative result for tumor markers after completion of the scheduled therapy. Of 46 patients, 36 patients achieved CR and 10 did partial response at best (Table 4). Of 36 subjects with CR, only 3 (8.3%) were RECQL1-high, while of 10 subjects without CR, 4 patients (40.0%) were RECQL1-high (Table 4, P <0.05), suggesting that the level of RECQL1 expression may be involved in chemo-resistance. However, no significant difference was found between the histological type and the rate of CR. Thus, the clinical studies have suggested that the expression of RECQL1 would elicit sensitivity of cancer cells to chemotherapy and elongate CR duration.

**Table 4:** **Relationship between chemo-sensitivity and RECQL1 expression**

**Response to chemotherapy**	**Number of patients**	**RECQL1- high expression**	**P value**
Complete response	36	3 (8.3%)	
Partial response	10	4 (40%)	< 0.05
Stable disease			
Progressive disease			

### Transfection efficiency of siRNA targeting RECQL1

**Figure 3:**
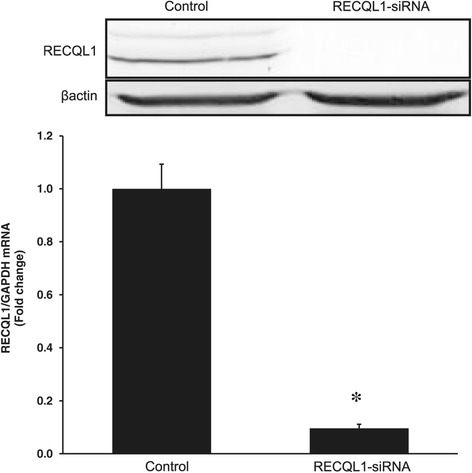
**Determination of RECQL1 mRNA expression in OVCAR-3 cells by RT-PCR after transfection of RECQL1-siRNA or control siRNA.** RECQL1 mRNA expression was significantly decreased in OVCAR-3 cells transfected with RECQL1-siRNA compared to those transfected with control siRNA. *P <0.001 vs. control. Western blot inserted here confirmed a disappearance of RECQL1 protein in RECQL1-siRNA cells.

In order to clarify the effects of RECQL1 on cancer cell growth, following in vitro studies were conducted. First, the transfection efficiency of RECQL1-siRNA was examined by determining the RECQL1 mRNA expression in OVCAR-3 cells. Expression of RECQL1 mRNA decreased significantly in OVCAR-3 cells transfected with RECQL1-siRNA (RECQL1(−) cells) compared to OVCAR-3 cells transfected with control siRNA (RECQL1(+) cells) (Figure 3, P <0.001). Western blot also showed a disappearance of RECQL1 protein in RECQL1(−) cells (Figure 3).

### Comparison of the growth of RECQL1(−) and RECQL1(+) cells

**Figure 4:**
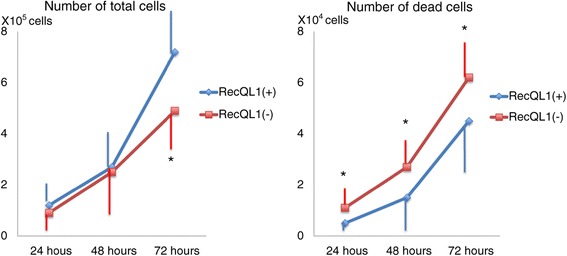
**Comparison of the number of total or dead OVCAR-3 cells at a time course of 24 hours to 72 hours after transfection.** OVCAR-3 cells (1 × 10^4^ cells) were cultured in 6-well plates. While the number of the total cells is significantly smaller in the RECQL1(−) cells than in the RECQL1(+) cells at 72 hours after transfection, the number of the dead cells is significantly greater in the RECQL1(−) cells than the RECQL1(+) cells at 24 to 72 hour after transfection. *P <0.05.

**Figure 5:**
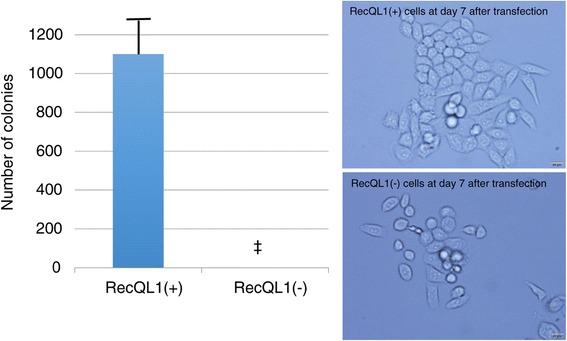
**Comparison of the number of colonies of OVCAR-3 cells at day 7 after transfection.** While colonies of RECQL1(+) cells increased, no colonies of 50 or more RECQL1(−) cells were found at day 7 after transfection (left figure). The photomicrographs show formation of colonies of RECQL1(+) and RECQL1(−) cells at 400x (right figure). ‡ P <0.0000 vs. RECQL1(+).

While the number of the total cells is significantly lower in the RECQL1(−) cells than in the RECQL1(+) cells at 72 hour after transfection, the number of the dead cells is significantly higher in the RECQL1(−) cells than the RECQL1(+) cells at 24 to 72 hour after transfection (Figure 4). In addition, while colonies of RECQL1(+) cells increased with time, no colonies of 50 or more RECQL1(−) cells were found at day 7 after transfection (Figure 5, left). RECQL1(+) cells formed colonies in a cobblestone fashion. RECQL1(−) cells were weakly linked and were in smaller clumps than RECQL1(+) cells (Figure 5, right).

### Comparison of apoptosis in RECQL1(−) and RECQL1(+) cells

Double staining methods with annexin V and PI clearly demonstrated that RECQL1(+) cells were more live and less apoptotic at 24 hours after transfection (P <0.05 and 0.01, respectively) (Table 5), suggesting that inhibition of the expression of RECQL1 induced apoptosis in in OVCAR-3 cells.

**Table 5:** **Results of fluorescent double staining of cell membranes and nuclei analyzed by flow cytometry**

**Fluorescent double staining**	**RECQL1 (+)**	**RECQL1 (−)**	**P value**
Annexin V(−)/PI(−) (Live cells)	9133 ± 93.0	8863 ± 121.3	< 0.05
Annexin V(+)/PI(−) (Apoptotic cells)	160 ± 10.0	427 ± 42.3	< 0.01

Results represent means ± SD. Experiments were repeated thrice.

### Comparison of cell cycle distribution in RECQL1(−) and RECQL1(+) cells

**Figure 6:**
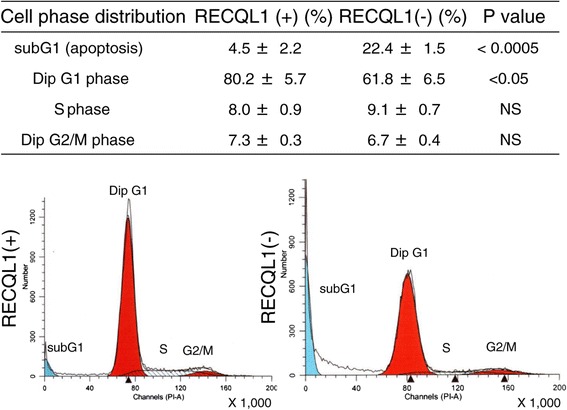
**Cell cycle distribution in RECQL1(+) and RECQL1(−) cells.** Cell cycle analysis showed a significantly greater distribution in subG1 phase (apoptosis) in RECQL1(−) cells than in RECQL1(+) cells. Results represent means ± SD. Experiments were repeated thrice. Dip; Diploid, NS: Not significant.

As shown in Figure 6, cell cycle analysis showed a significantly greater distribution in subG1 phase indicating apoptotic cells in RECQL1(−) cells than in RECQL1(+) cells. (P <0.0005). A significant difference was not found in G2/M phase between RECQL1(−) and RECQL1(+) cells, suggesting that inhibition of the expression of RECQL1 induces apoptosis but does not increase G2/M phase in which mitotic arrest (mitotic catastrophe) occurs.

## Discussion

Although significant association was not found between levels of RECQL1 expression and clinical background such as histological type, clinical stage, and retroperitoneal lymph node metastasis, the results of the present study have shown a close relationship between the recurrence of EOC and the level of RECQL1 expression. In addition, PFS of patients with RECQL1-high EOC was significantly shorter than that of RECQL1-low patients at stage III/IV EOC. Previous studies have shown that RECQL1 is expressed at high levels in cancer cells with rapid growth, while RECQL1 is expressed at low levels in cells at resting stage [18]. In addition, the higher level of expression of RECQL1 is shown in hepatic cancer at high grade [13]. Because non-malignant liver tissue has slight staining, it is proposed that RECQL1 can be a molecular marker that predicts malignancies and progression of liver cancer [13]. Sanada et al. demonstrated that high expression of RECQL1 is correlated with a high Ki-67 labeling index and that it can be a marker of high proliferation in ovarian cancer tissue [15]. Although we did not examine the relationship between expression of RECQL1 and cell growth such as Ki-67 labeling index in our subjects, we showed that high expression of RECQL1 was correlated with a significantly shorter PFS in stage III/IV EOC. Recurrence is a critical issue that worsens prognosis. Therefore, determination of RECQL1 can be a useful marker for planning strategy of tailor-made treatment.

The present study showed a close relationship between a high level of RECQL1 expression and resistance to chemotherapy in stage III/IV EOC. All patients with EOC in this study received combined chemotherapy with TC after surgery. Zhang et al. have shown that RECQL1 is expressed at high levels in cisplatin-resistant cancer cell lines derived from moderately-differentiated tongue squamous cell carcinoma, and they proposed that RECQL1 may be associated with the acquisition of cisplatin resistance [19]. The formation of DNA adducts by cisplatin results in apoptosis, and several studies have shown that apoptosis is attenuated in cells with extensive DNA repair [20,21]. Although the EOC patients used in this study were treated with carboplatin, a platinum analogue like cisplatin, expression of RECQL1 is shown to be involved in chemo-resistance and the results of present study support previous findings that high expression of RECQL1 attenuates the effectiveness of platinum analogues [19].

In order to clarify the biological significance of RECQL1 expression in cell growth, RECQL1 siRNA was transfected into OVCAR-3 cells. As shown in Figures 4, 5, and 6 and Table 5, RECQL1 siRNA transfected-cells significantly reduced cell growth and increased apoptosis compared to those of control cells. An earlier study showed increased mitotic death (mitotic catastrophe) was prominent in a number of cancer cells transfected with RECQL1 siRNA [12]. Mitotic catastrophe occurs once the cell division cycle that is not repaired by RECQL1 reaches the M phase. In addition, if cancer cells expressing high levels of DNA repair enzymes such as RECQL1 are affected by specific and rapid silencing via RECQL1 siRNA, cancer cells retaining DNA abnormalities proceed to the M phase of the cell division cycle and mitotic arrest (mitotic catastrophe) occurs in the M phase. However, the present study did not show an increased cell distribution of the M phase in which mitotic catastrophe occurs in RECQL1 siRNA transfected-cells. It is suggested in this study that OVCAR-3 cells with decreased RECQL1 expression yielded to apoptosis. Arai et al. reported that transfection of RECQL1 siRNA in the presence of cisplatin caused apoptosis of cancer cells [14]. Ngan et al. indicated that the anticancer drug oxaliplatin causes both mitotic catastrophe and apoptosis [22]. Although the mechanisms of cell death, including apoptosis, mitotic catastrophe, and necrosis, still need to be elucidated [23], results of the present study suggest that silencing RECQL1 induces apoptosis.

In earlier studies, reduction of RECQL1 expression in cancer cells through using siRNA showed an inhibitory effect on growth of cancer in vivo [12,24]. In contrast, normal cells were not affected by the silencing of RECQL1 [12]. This fact may signal a breakthrough for a new strategy to treat malignant tumors including ovarian cancer. Induction of apoptosis is an important part of designing anticancer drugs. RECQL1 induces apoptosis, so drug discovery targeting RECQL1 is a promising area for future research.
